# Rice Husk-Derived Cellulose Nanofibers: A Potential Sensor for Water-Soluble Gases

**DOI:** 10.3390/s21134415

**Published:** 2021-06-28

**Authors:** Naresh Shahi, Eunji Lee, Byungjin Min, Dong-Joo Kim

**Affiliations:** 1 College of Agriculture, Environment and Nutrition Sciences, Tuskegee University, Tuskegee, AL 36088, USA; nshahi1164@tuskegee.edu; 2 Department of Mechanical Engineering, Materials Research and Education Center, Auburn University, Auburn, AL 36849, USA; ezl0020@auburn.edu; 3 Department of Food and Nutritional Sciences, Tuskegee University, Tuskegee, AL 36088, USA

**Keywords:** cellulose, nanocellulose, agricultural byproducts, gas sensor, biopolymers

## Abstract

Cellulose and its derivatives have evoked much attention in sensor technology as host-matrices for conducting materials because of their versatility, renewability, and biocompatibility. However, only a few studies have dealt with the potential utilization of cellulose as a sensing material without a composite structure. In this study, cellulose nanofibers (CNF) and 2,2,6,6-tetramethylpiperidine-1-oxyl (TEMPO)-oxidized cellulose nanofibers (TOCNF) extracted from rice husks by using ultrasonic-assisted methods are introduced as a potential gas sensing material with highly sensitive performance. To fabricate nanocellulose-based films, CNF, TOCNF, and TOCNF with glycerol (TOCNF/G) were dispersed in water and applied on polyimide substrate with digital electrodes to form self-standing thin films by a drop-casting method. A transparent coating layer on the surface of the plate after drying is used for the detection of water-soluble gases such as acetone, ammonia, methane, and hydrogen sulfide gases at room temperature at 52% relative humidity. The sensor prototypes exhibited high sensitivity, and the detection limit was between 1 ppm and 5 ppm, with less than 10 min response and recovery time. The results indicate that both the CNF- and the TOCNF-coated sensors show good sensitivity toward ammonia and acetone, compared to other gases. A TOCNF/G-coated sensor exhibited minimum time in regard to response/recovery time, compared to a CNF-coated sensor. In this study, nanocellulose-based sensors were successfully fabricated using a low-cost process and a bio-based platform. They showed good sensitivity for the detection of various gases under ambient conditions. Therefore, our study results should further propel in-depth research regarding various applications of cellulose-based sensors in the future.

## 1. Introduction

Cellulose and its derivatives have been considered as substrates for green sensing materials because they are cost-effective, light-weight, flexible, sustainable, and renewable [1,2]. Cellulose, especially cellulose nanofibers (CNF) and cellulose nanocrystals (CNC), have unique properties such as high Young’s modulus, transparency, and high-temperature resistance [3]. Cellulose can be modified into different structures by chemical methods, and these modified forms have fascinating applications in flexible and disposable sensors [2]. Cellulose is porous, and it has a hygroscopic network consisting of intertwined fibers in which hydroxyl groups exist on its molecular chains [4]. Dry cellulose paper contains around 5% moisture by weight at 50% relative humidity [5]. The moisture on the surface of cellulose could be a medium to dissolve water-soluble analytes, and the surface can act as a charge carrier to generate currents. Thus, this study aims to utilize the inherent properties of cellulose to detect water-soluble gases.

In general, rice husks generated from the milling process are considered as a waste and low-value agricultural byproduct [6]. They are mainly composed of materials such as cellulose, lignin, and silica. The concentrations of cellulose and silica range from 35–50 wt.% and 15–28 wt.%, respectively [7]. Cellulose fibers can be converted into CNF and or CNC by different methods such as mechanical, chemical, enzymatical, and/or a combination of a mechanical and a chemical process [8]. Among the mechanochemical methods, 2,2,6,6-tetramethylpiperidine-1-oxyl (TEMPO)-mediated oxidation and the ultrasonic-assisted method have become widespread due to the ease-to-use and efficient production of nano-sized cellulose [9,10,11]. The chemical and physical properties of CNF produced via TEMPO-mediated oxidation depend on the carboxyl content and reaction conditions. The introduction of charged groups onto the surface of cellulose is a fast and effective way of CNF production [12,13,14]. Also, TEMPO-mediated oxidation is a promising method to effectively modify the surface of native cellulose. Compared to unmodified CNF, TEMPO-oxidized CNF presents carboxylic groups on the surface of cellulose making it negatively charged and increasing the degree of fibrillation; thus, water-soluble and optically transparent films can be obtained [15,16].

Various sensors have been developed to detect volatile molecules, such as polymer-based chemoresistive sensors, 2D sensors, and inorganic metal oxide sensors [17,18]. Among these, inorganic metal oxide sensors are promising due to their thermal and chemical stability in the air. The sensing mechanism in metal oxide sensors is mainly based on electron transfers between the target gas and the sensor material surface [19]; however, long-term exposure tends to deteriorate the sensing performance and cause environmental problems [20]. Biopolymer-based sensors such as nanocellulose and its composites might effectively address the problems associated with the low performance of metal oxide-based sensors for long-term use because cellulose is a highly sustainable as well as environmentally friendly material. From this perspective, nanocellulose-based sensors can represent an alternative green technology in the sensor industry. For instance, a composite of carbon nanotube and cellulose can be used as a humidity sensor [21]. Primarily, nanocellulose is utilized as a carrier substrate and template in a conducting composite structure. It is reported that polyaniline/polyvinyl alcohol with carbon nanofiber and nano-fibrillated cellulose can be used in a sensor for monitoring humidity and ammonia gas [22]. Cellulose can also be hybridized with metal oxides to develop composite membranes or thin films for H_2_S gas sensing [23,24]. A cellulose acetate-based polymeric membrane detects 1 ppm of ammonia with a fast response (60 s) and recovery (78 s) time [25]. Similarly, cellulose paper can detect multiple gases such as CO, CO_2_, H_2_S, and NH_3_; it is the most sensitive towards ammonia with a fast and reversible response [5]. Recently, emerging trends in green technology also focus on sustainable, degradable, and cost-effective sensing materials that are highly functional under ambient conditions. In this line of investigation, this study reports the use of rice husk-derived CNF-based films as potential gas sensors to detect water-soluble gases at room temperature without doping.

## 2. Materials and Methods

### 2.1. Chemicals and Materials

Sodium bromide (NaBr), 2,2,6,6-tetramethylpiperidine-1-oxyl (TEMPO), sodium hypochlorite acid (NaClO solution, available chlorine 7–10%), glycerol (100%), hydrogen peroxide (30% containing inhibitors), ethanol, and acetone were purchased from Sigma Aldrich (St. Louis, MO, USA). Sulfuric acid and sodium hydroxide were also purchased from Sigma Aldrich. Rice husk (RH) was purchased from Three H’s, LLC (Crossett, AR, USA). RH ground powder was further disintegrated in a ball mill for 2 h to form a fine powder. The milled powder samples were sieved using 50 µm diameter mesh to select homogeneous particles for further analysis.

### 2.2. Extraction of Cellulose Fibers/Nanofibers

Previously extracted cellulose fibers and cellulose nanofibers from rice husks in the laboratory were used to fabricate gas sensing devices. Details of the extraction methodology can be found in our previous studies [26,27]. In brief, homogeneous dried RH powder was hydrolyzed using mild sulfuric acid (0.1 M) with continuous stirring at 75 ± 5 °C for 2 h. The solution was filtered and washed with distilled water until the filtrate reached neutral pH. The remaining pulp was hydrolyzed in alkaline hydrogen peroxide (AHP) solution at high pH (~11.05) in an ultrasonic bath for 2 h at 50 ± 1 °C. The undissolved solid portion contained cellulose pulp. The pretreated cellulose pulp was washed several times with deionized water until the filtrate reaches to neutral pH, followed by oven drying to produce cellulose fibers. Extracted cellulose fibers were the source of nanocellulose produced using the ultrasonic-chemical method. Details of the methodology and morphology of the synthesized cellulose can be found in a previous study [26]. In brief, cellulose pulp was irradiated using an ultrasonic titanium horn (frequency of 20 kHz for 2 h) and the obtained slurry was centrifuged at 10,000 rpm for 10 min at 4 °C to further remove water, then dried in an oven at 60 °C for 12 h for further characterization. In this study, the extracted cellulose nanofibers were named CNF.

### 2.3. Cellulose Modification/TEMPO-Oxidation

Cellulose fibers (AHP-treated) from rice husks used for CNF production were used to modify cellulose using TEMPO-mediated oxidation. One gram of cellulose fibers was dispersed in 100 mL of deionized water. A solution of TEMPO (0.012 g) and NaBr (0.124 g) was added into the cellulose suspension. Then 5 mL of 10–15% sodium hypochlorite (NaClO) solution was added dropwise to start the reaction. The suspension was continuously stirred at room temperature for 2 h at pH 10 ± 0.2, then the pH was adjusted with 0.4 M NaOH solution (monitored with a pH meter). After the designated reaction time, cellulose suspension was thoroughly washed in deionized water using a vacuum filter. The remaining pulp was further treated under an ultrasonic probe for 10 min in an ice bath to obtain the TEMPO-oxidized cellulose (TOCNF) slurry. The reaction was terminated by adding 5 mL of 70% ethanol. The TOCNF suspension was purified using a centrifuge and washed with deionized water until a neutral pH was achieved. This process transforms cellulose fibers into nanoscale oxidized cellulose.

### 2.4. Gas-Sensing Sample Preparation

A simple drop-casting method was utilized to fabricate the cellulose-based gas sensor devices. Flexible polyimide film is used as sensor substrate and nanocellulose suspension prepared in deionized water was coated on its surface. Polyimide film was cleaned by ultrasonication with 99.9% liquid ethanol and subsequently with deionized water, and then any remaining moisture was completely blown off using argon. The arrays of interdigitated electrode patterns engraved on a stainless-steel mask with 400 interdigitated gaps between electrodes were taped on the clean polyimide film. Direct current (DC) sputtering (Discovery 18, Denton Vacuum, Moorestown, NJ, USA) was employed with a platinum target (99.9%, Lasker, Jefferson Hills, PA, USA) on the masked substrate under argon gas after a pre-sputtering step to clean the surface of the target. To ensure uniform deposition, the rotation speed was set to 50 rpm, and the thickness of the obtained platinum electrode was measured to be ~100 nm.

### 2.5. Selection of Sensing Material

Different films such as rice husk (raw), cellulose fibers, cellulose nanofibers derived from rice husks (CNF), and TEMPO-oxidized cellulose (TOCNF) were prepared as sensing materials. All the films consisted of ~0.1 wt.% of dry cellulose/nanocellulose dispersed in deionized water. The suspension was continuously heated by stirring at 250 rpm at 80 °C for 30 min. A drop of each suspension was gently deposited onto a pair of platinum electrodes on the polyimide substrate using the 5 mL capacity disposable pipet. A droplet of the uniformly dispersed suspension was then air-dried, placing under the hood for 10 to 12 h at room temperature, and stored in a desiccator for further analysis. The film thickness was measured by a digital caliper (Traceable^®^, S/N 192161251, Thomas Scientific, Candler, NC, USA), and each value was expressed as an average of five representative measurements. The thickness of the deposited films was ranged from ~30 to ~35 µm. Samples with initial conductive signals between ~50 KΩ to ~10 MΩ in voltameter (WH5000A, AstrolAL, Placentia, CA, USA) were selected for further analysis as gas sensor devices. TOCNF exhibited very high resistance; therefore, glycerol (0.03 g) was added to modify the TOCNF. The TOCNF suspension was vigorously stirred for 30 min for homogenous dispersion of glycerol and named TOCNF/G. After the addition of glycerol, the TOCNF/G sensor device significantly reduces the resistance to ~5 MΩ. Therefore, only two sensors from CNF and TOCNF/G were selected for further evaluation and compared in the study.

### 2.6. Gas-Sensing Device Fabrication

A fully automated in-house designed sensing apparatus was applied to evaluate the sensing properties of the samples with the selected target gases. Two conductive pads of the interdigitated electrode were connected to resistance measurement equipment (mod. 2400 source meter, Keithley, Beaverton, OR, USA) to extract the electrical signals. The sensing materials resistance change was recorded in the presence or absence of the target gas under continuously blowing synthetic air within the chamber by a custom-designed LabVIEW software routine. Two types of dry gas, i.e., oxygen and nitrogen, were mixed to produce synthetic air in a ratio of 80:20; the amount was controlled by a mass flow controller (MKS1179A, MKS Instruments, Andover, MA, USA). To inject the target gas, the total gas flow was fixed to 100 sccm by combining three gases; pure nitrogen (MFC 1), pure oxygen (MFC 2), and target gas diluted in nitrogen (MFC 3). The amount of oxygen was fixed to 20 sccm during the synthetic air and analyte injection, and the amount of pure nitrogen and analyte was altered with an analyte concentration by the following equation:

$$C_{\mathrm{analyte}}\left(\mathrm{ppm}\right)=\frac{C_{\mathrm{gas}}*\mathrm{MFC}\;3}{\mathrm{MFC}\;1+\mathrm{MFC}\;2+\mathrm{MFC}\;3}$$

To adjust 100 ppm of acetone gas concentration ($C_{\mathrm{analyte}})$, 60 sccm of pure nitrogen (MFC 1), 20 sccm of pure oxygen (MFC 2), and 20 sccm of acetone-diluted nitrogen (MFC 3), with the 500 ppm concentration ($C_{\mathrm{gas}}$) were injected. Before the exposure of target gas, the cellulose sensors were flushed with synthetic air to stabilize the nanocellulose films for at least 30 min. The target gas was then introduced into the sensing chamber with an alternative cycle of 10 min on/off at room temperature by changing the target gas concentration.

### 2.7. Sensor Characterization

Crystallinity was analyzed at room temperature using an X-ray diffractometer (D8 Discover, Bruker, Billerica, MA, USA) equipped with LYNXEYE^TM^ with monochromatic CuKα radiation (40 kV and 40 mA) source (λ = 0.154 nm) in the step-scan mode with a 2θ angle/minute ranging from 5° to 50° with a step of 0.01. The chemical structure of the samples was analyzed by Fourier transform infrared (FTIR) spectroscopy. Samples were placed on the universal attenuated total reflectance (ATR)-FTIR equipped with silver gated zinc selenide crystal (Nicolet, Thermo Electron Corporation, WI, USA) at room temperature. The experiments were carried out in the range of 650–4000 cm^−1^ with the resolution 4 cm^−1^, and total scans were 32 per sample. Surface morphology and the sample’s dimension were evaluated on A JEM-JSM-7200F model field emission-scanning electron microscope (FE-SEM, JEOL USA, Peabody, MA, USA). Samples were mounted on conductive adhesive carbon tape and sputtered with gold/palladium target for 5 min. During sputtering, plasma discharge current was maintained below 10 milliamperes and observed under the FE-SEM using a 10.0 kV.

## 3. Results and Discussion

### 3.1. Cellulose and Nanocellulose Extraction from Rice Husks

**Figure 1:**
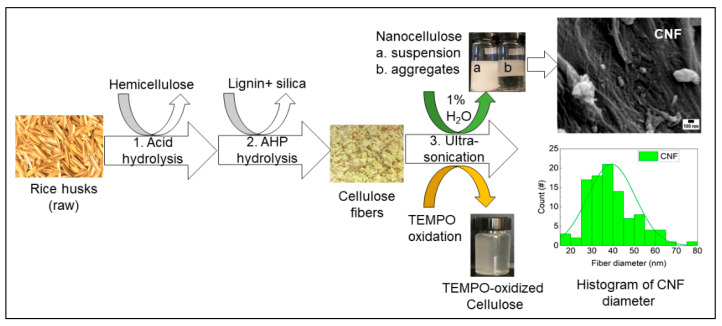
Fractionation of lignocellulose and silica from rice husks (RH). Extraction of cellulose nanofibers (CNF) and TEMPO-oxidized cellulose from rice husks using an ultrasonic-assisted method.

The three-step extraction process of cellulose, hemicellulose, and lignin from rice husks is presented in Figure 1. The lignocellulosic composition of cellulose, hemicellulose, and lignin from rice husks (raw) was around 35%, 19%, and 22%, respectively. In the first step, hemicellulose was dissolved using diluted sulfuric acid and removed by vacuum filtration. In the second step, the remaining pulp was dissolved in alkaline hydrogen peroxide (AHP) to remove lignin and silica, and this step also removed the remaining hemicellulose fraction. The undissolved white pulp obtained was cellulose, a dried form of white fiber (see Figure 1), and the concentration of cellulose fiber was around 62%. Finally, obtained cellulose fibers were further treated with 1% H_2_O_2_ solution and TEMPO-oxidation separately using an ultrasonic probe. The resultant, gel-like suspension of cellulose slurry was produced after ultrasonication with 1% H_2_O_2_. In contrast, the transparent suspension was produced with TEMPO-oxidized cellulose (see Figure 1). The composition of the lignocellulosic components of RH and the morphological properties of fibers (nanoscale) can be found in our previous study [27].

It is reported that the hemicellulose from sugarcane bagasse decreased from 24.5% to 7.8% after sulfuric acid (1%) treatment, and further treatment with sodium hydroxide (1% or higher) removed 96% of hemicellulose [28]. Hydrogen peroxide in AHP solution promotes delignification and removal of any remaining hemicellulose by its oxidative action, resulting in almost pure cellulose fibers [29]. The alkaline conditions break the intermolecular ester bond between lignin and carbohydrates; thus, compact structure relaxed and dissolved lignin [29,30]. Ultrasound impacts the physical and chemical characteristics of biomass, such as delignification, reduce particle size, and increase surface area [31].

### 3.2. TEMPO-Oxidation

Cellulose fibers were extracted using TEMPO-oxidation and modified with glycerol. TEMPO-oxidation process can be converted C6-OH groups of cellulose to sodium C6-carboxylate group through C6-aldehyde [16]. Surface modification of the cellulose can be used as new alternative substrates for various applications, such as printed electronics [32]. The reaction uses either NaClO or NaClO_2_ as an oxidizer in the presence of NaBr in alkaline conditions, and its analogs are water-soluble. One of the main advantages of CNF synthesized through this methodology is high optical film transparency that carries carboxylic groups [15]. At higher relative humidity, TEMPO-oxidized cellulose nanofibers films increase the oxygen permeability due to hydrophilic, but dried films decrease in oxygen barrier properties [16].

### 3.3. General Characteristics of Nanocellulose and Sensors

**Figure 2:**
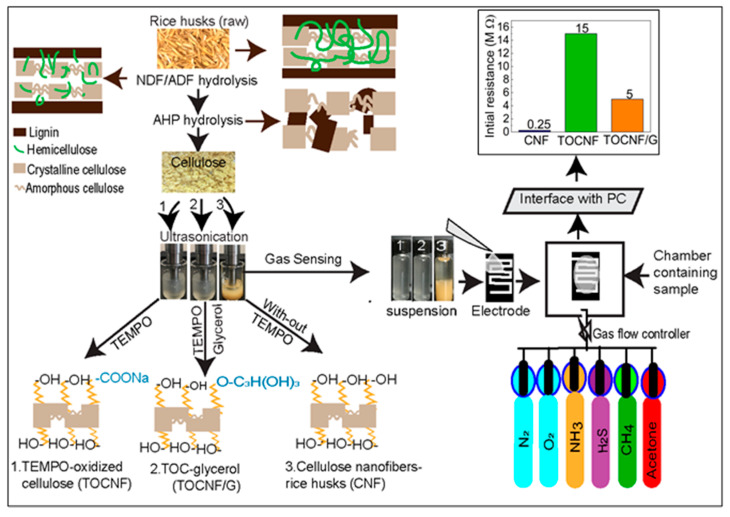
Schematic representation of cellulose nanofibers and TEMPO-oxidized cellulose modification process from rice husks (**left**) and fabrication of self-standing nanocellulose film from extracted cellulose nanofibers (CNF), TEMPO-oxidized cellulose (TOCNF), and TOCNF with glycerol (TOCNF/G) against various water-soluble gases with their initial resistance (**top right**).

The extraction process of the cellulose nanofibers is illustrated in Figure 2. In our study, all samples have different surface structures in which the extracted cellulose nanofibers (CNF) possess three possible -OH groups available for hydrogen bonding. However, TOCNF has carboxylic acid groups and -OH groups and the addition of glycerol in TOCNF increased -OH groups. The schematic representation of surface structure with functional groups for CNF, TOCNF, and TOCNF/G are presented (see Figure 2, left). Homogeneous nanocellulose slurry obtained from rice husks was used to fabricate a thin self-standing film. The initial resistance of prepared film with CNF and TOCNF was ~0.25 MΩ and ~15 MΩ, respectively. The TOCNF was modified with glycerol, and the measured resistance of TOCNF/G was found drastically decreased to ~5 MΩ (see Figure 2, top right). During TEMPO-oxidation, only the hydroxymethyl groups of cellulose were oxidized, while secondary hydroxyl remains unaffected due to this topologically confined reaction sequence, only half of the accessible hydroxymethyl groups are available to react [3]. It means TOCNF does not have sufficient -OH ions to interact with water, which obviously has less ionic conductivity because fewer water-soluble analytes could interact with it; thus, it might be why electric resistance of the TOCNF sensor was very high (~15 MΩ). Therefore, TOCNF was further treated with glycerol to increase the -OH ions on cellulose surface. As expected, the initial resistance of the TOCNF/G sensor was decreased, and it was around 5 MΩ at room temperature, which is a noticeably lower range than the TOCNF sensor. Since the TOCNF exhibited high resistance, further analysis was only performed on CNF and TOCNF/G films.

It is reported that a composite of TEMPO-oxidized cellulose fibers and poly (ethylene glycol) was fabricated as a humidity sensor and the sensor exhibited a significant change of impedance in response to relative humidity from 20% to 90% [33]. In another study, a cellulose-based sensor impregnated with a palladium complex and ethylene glycol increased the response to H_2_S by four-fold compared to the sensor without the ethylene glycol [34]. The ethylene glycol acts as a humectant, facilitates solid/gas phase interaction by increasing the number of water molecules on the film surface, and enables a higher input of gas molecules on the cellulose layer [35]. Glycerol is an organic compound that consists of a three-carbon chain with -OH groups attached to each carbon and a similar structure to ethylene glycol. Therefore, we expected that glycerol addition might increase the absorption of water molecules on the surface of CNF. FTIR analysis confirmed the presence of OH groups after glycerol incorporation in TOCNF as discussed in the following section.

### 3.4. XRD and FTIR Analysis

**Figure 3:**
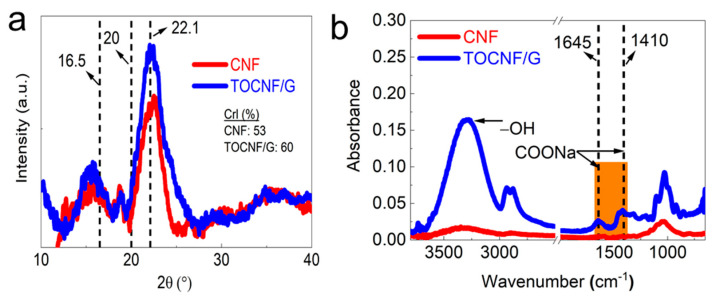
(**a**). XRD peaks and (**b**). FTIR spectra of CNF and TEMPO-oxidized cellulose with glycerol (TOCNF/G).

XRD and FTIR characterized the crystallinity and chemical structure of CNF and TOCNF/G (see Figure 3a,b). The results showed distinct and well-defined peaks at around 16.5, 20, and 22.1 at 2θ (see Figure 3a). It was reported that these peaks correspond to crystalline cellulose [36,37]. As expected, TOCNF displayed a higher percentage of crystallinity compared to TOCNF/G and CNF (Supporting Information, Table S1). The increased crystallinity by TEMPO-oxidation has resulted from the progressive removal of amorphous-cellulosic substances [38]. Generally, the crystallinity can influence the structure and property of materials. The smaller crystal size of bacterial cellulose increases its dispersibility into a metal nanoparticles matrix, leading to excellent sensor ability towards hydrogen sulfide [23]. CNF exhibited other peaks at different 2θ degrees compared to the peaks mentioned above, probably due to a small portion of silica in the CNF. The corresponding EDS spectrum examined the elemental composition of the CNF, and the result is available from our previous study [27], which confirmed that the presence of silica in CNF. The broad peak at 2θ = 22° was assigned for amorphous silica in rice husk was reported [39].

The FTIR analysis verified the surface modification of cellulose with TEMPO-oxidation, changes in chemical structure with glycerol, and the presence of hydroxyl groups. The spectra intensity was compared between CNF and TOCNF/G, as shown in Figure 3b. Dominant peaks were observed at around 3500 and 3000 cm^−1^ due to OH-stretching and CH- stretching, respectively. TOCNF/G exhibited a sharper peak in this region, which was more intense and prominent than CNF (see Figure 3b). It might be due to glycerol, which provides more -OH group in the films. Comparative FTIR peaks of CNF, TOCNF, and TOCNF/G are also available (Supporting information, Figure S1). In TOCNF/G films, prominent peaks were found at around 2875 and 2950 cm^−1^ due to symmetric and asymmetric vibrations of the methyl group [40,41]. The changes in the spectrum of TOCNF/G compared to CNF in this region might be due to surface modification with TEMPO-oxidation or the addition of glycerol because these peaks were observed in TOCNF and TOCNF/G. The most crucial difference is the carboxyl group’s (C=O) stretching band appearance at around 1645, 1602, and 1410 cm^−1^ in TOCNF/G films, indicating that hydroxyl groups at the C6 position of cellulose molecules are converted to sodium carboxylate (-COONa) [12,42]. In addition, the pristine TOCNF shows a prominent peak at around 1596 cm^−1^, corresponding to carboxylate ions (COO^-^) [43]. All the peaks mentioned above are present only in TEMPO-oxidized cellulose. 

Similarly, samples exhibited sharper peaks at around 1300, 1155, and 1060 cm^−1^. These peaks are associated with the C-O stretching and CH- deformation vibrations of cellulose. Regardless of the samples, the peak intensity around 1030, 1060, 1160, and 2923 cm^−1^ is prominent, where these peaks are associated with cellulose. The region of 1500 and 1600 cm^−1^ is related to a vibration band of aromatic compounds of either acetyl and uronic ester groups of hemicellulose or ester groups of the ferulic groups of lignin peak [44]. The results indicated the absence of peaks for this reason, which implies the successful removal of lignin and hemicellulose from the samples with the applied method.

### 3.5. Surface Analysis of Gas Sensors

**Figure 4:**
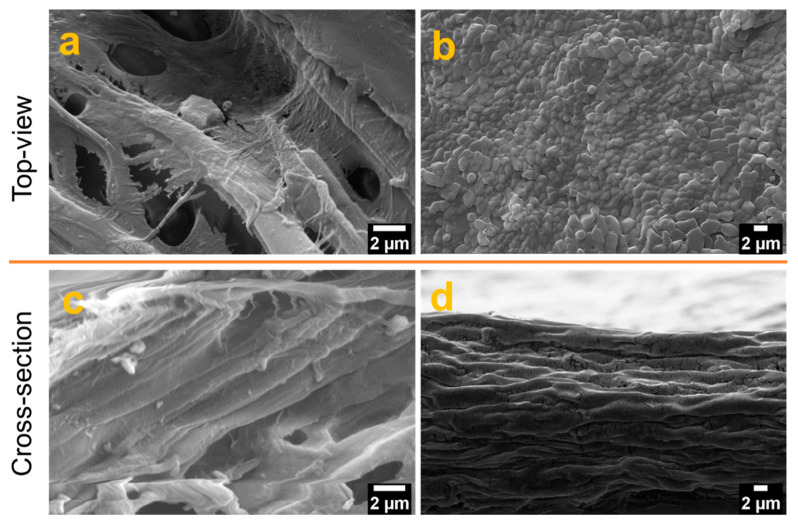
Film morphology at different magnification by FE-SEM: top view (top) images (**a**) (12,000×) and (**b**) (5000×), and cross-section view (bottom) (**c**) (16,000×) and (**d**) (5000×) of CNF, and TOCNF/G, respectively.

Field emission-scanning electron microscopy (FE-SEM) was used to analyze the surface morphology of the films from the top- and cross-section area and the results are presented in Figure 4. The top-surface topography of the film TOCNF/G was relatively smooth compared to CNF, and CNF surface was porous (see Figure 4a,b). 

A cross-section image of CNF film possesses heterogeneous network (see Figure 4c). However, the cross-section view of the TOCNF/G shows layers of long homogeneous fibers tightly stacked with each other in a bundle structure (see Figure 4d). The difference in surface morphology between CNF and TOCNF/G film may affect the sensors’ gas response properties. At a particular relative humidity, moisture absorption on cellulose fibers depends on the microstructure, porosity, and the portion of the chemical composition [45]. Besides, the rough and porous structure of cellulose creates a large interfacial area that can immobilize functional molecules. This property improves the interaction between surface and water vapor molecules and, thus, a suitable substrate for microfluidic transportation from the capillary force [46,47].

### 3.6. The Role of Absorbed Water on Sensor

**Figure 5:**
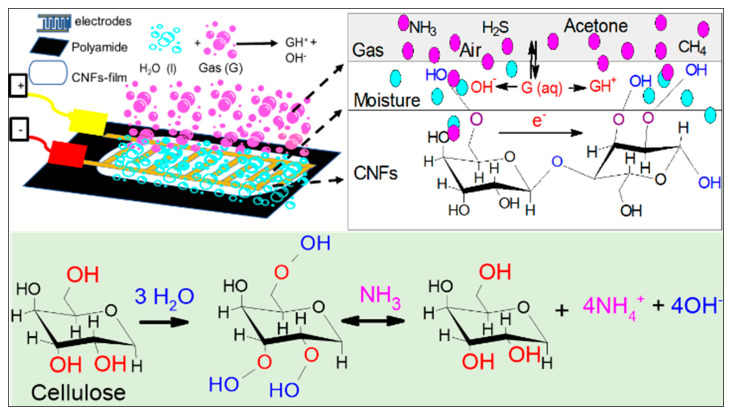
Schematic representation of possible water-soluble gas sensing mechanism in CNF-film surface.

Since cellulose has an inherent moisture absorption property due to the freely available hydroxyl groups that interact with water molecules, a solvation cage is formed and a monomolecular layer of water develops [48]. The interaction of the hydroxyl groups of cellulose and water creates a single electron cloud and becomes a single entity. Water absorbed on cellulose nanofibers partially dissolves water-soluble gases such as ammonia and dissociates ammonia into ammonium (NH_4_^+^) and hydroxide (OH^−^) ions (Figure 5). The dissociation of ammonia in water elevates the ionic concentration, which can be detected through electrical measurements, resulting in a decrease in the sensor’s electric resistance with increase ionic concentration and vice versa. For instance, in the absence of water-soluble gases, chemical reaction will be reversed, resulting in increased sensor resistance to its original value as shown in the following reaction:

$${\mathrm{NH}}_{3}+H_{2}O↔{\mathrm{NH}}_{4}^{+}+{\mathrm{OH}}^{-}$$

The increased conduction due to the dissociation of target gas depends on ionic mobility, charge, ions concentration, and water solubility [5]. Similarly, when the sensors produced are exposed to H_2_S, the oxygen molecules absorbed interact with H_2_S gas and trap them at the surface in the form of ions as shown in the following reaction [49]:

$$2H_{2}S+3O_{2}^{-}\;\left(\mathrm{adsorb}\right)=2H_{2}O+2{\mathrm{SO}}_{2}+3e^{-}$$

Cellulose sensors require moisture for operation, and cellulose sensors can rapidly absorb and desorb water molecules with the changing environmental humidity, resulting in a change of output resistance. Also, a response is very fast with increased % relative humidity. At a higher percentage of relative humidity, more water molecules are readily available for interaction, and their adsorption to the sensor surface is relatively fast. However, it took more time to reach a saturated value [50]. It is reported that high sensitivity humidity sensors can be fabricated with nanofibrillated cellulose with graphene oxide and polydimethylsiloxane; however, the desorption curve of the sensor lagged behind its adsorption curve in the range of 75% to 85% relative humidity [51]. In another study with cellulose/carbon nanohorn sheets, the sheets’ resistance before and after exposure to water vapor resistance was decreased in a dry sheet; however, the resistance has gradually increased with the increase in the water vapor % (relative humidity) was reported [47]. Besides, cellulose nanofibers (CNF) are prone to swelling when CNF/carbon nanotube composite humidity sensors are exposed to humidity conditions, causing a shape decrease in electrical conductance because swelling CNF disrupts the carbon nanotube conductive networks under high relative humidity [21]. In contrast, cellulose sensors may not function with sufficient high performance in an environment with low relative humidity (<20%) [5]. At constant relative humidity, the presence of water vapor leads to an increase in the NH_3_ response, as moisture acts as a reaction catalyst or involves a reaction mechanism with NH_3_ in a supportive manner [52]. We analyzed the gas sensing properties of the sensors at constant relative humidity (52%) throughout the experiment at room temperature.

**Figure 6:**
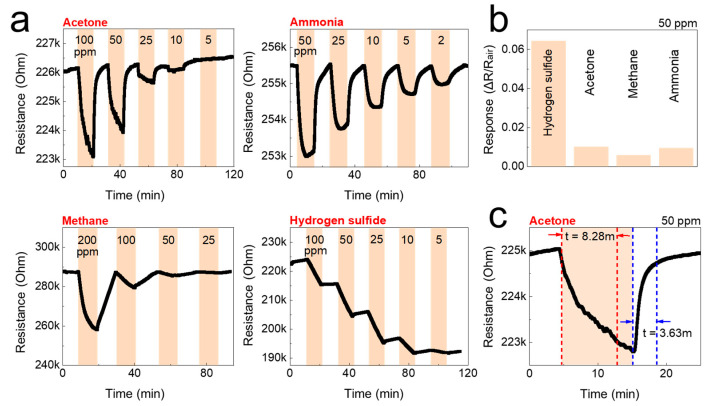
Gas-sensing performance of the CNF sensor against acetone, ammonia, methane, and hydrogen sulfide at room temperature. (**a**) Real-time resistance behavior of the CNF sensors as a function of the concentration of the test gases; acetone, ammonia, methane, and hydrogen sulfide. (**b**) The gas response of the CNF sensors to 50 ppm of the test gas. (**c**) Gas response time of the CNF sensors to 50 ppm of acetone.

**Figure 7:**
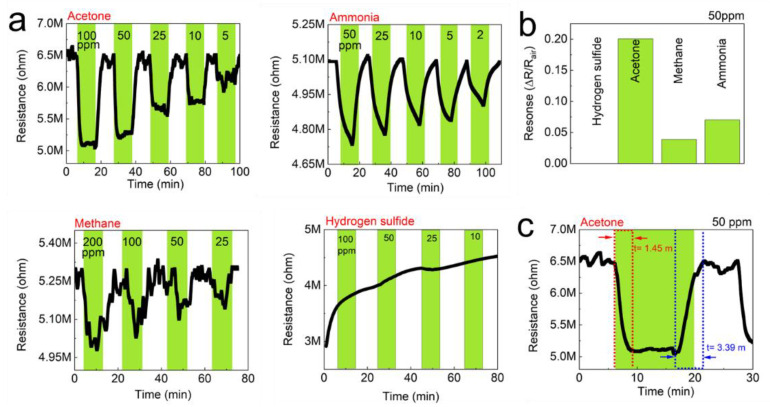
Gas-sensing performance of the TOCNF/G sensors against acetone, ammonia, methane, and hydrogen sulfide at room temperature. (**a**) Real-time resistance behavior of the TOCNF/G sensors as a function of the concentration of the test gases; acetone, ammonia, methane, and hydrogen sulfide. (**b**) Gas response of the TOCNF/G sensors to 50 ppm of the test gases, (**c**) Gas response time of the TOCNF/G sensors to 50 ppm of acetone.

In our study, the FTIR results verified the presence of hydroxyl groups on the sensors, and the concentration of hydroxyl groups was more in TOCNF/G films than CNF ones. Based on this result, the concentration of NH_3_ might be higher on TOCNF/G than that of CNF, making the TOCNF/G sensor more sensitive and giving a higher response than the CNF sensor. The result is verified by comparisons between TOCNF/G and CNF sensors response towards study analysts (see Figure 6b and Figure 7b). The findings indicate that the surface structure could also influence the sensors’ sensing performance because CNF was more porous and less crystalline than TOCNF/G, as confirmed by SEM and XRD analysis. Although there is no definitive answer to the gas sensing mechanism of nanocellulose, our proposed mechanism can be supported by a similar consensus on studying a thin film of water in the interconnected network of cellulose that reacts with the gas molecules modulates cellulose conductivity [2,4,5]. 

From this interpretation, we believe that the cellulose surface functional groups and surface morphology could be instrumental in responding to water and water-soluble gases, thus influencing the ionic conductivity and responses towards water-soluble gases. The responses of sensors towards different gases concentration were measured to support this mechanism, and the results are discussed in the following section.

### 3.7. Gas Sensing Properties

The detection of gas sensors is based on the resistivity change of the materials. The resistance of the studied sensors decreased upon exposure to the tested analytes (gases), and recovered to its initial value when gases were replaced by air. The response/recovery curves to analytes of CNF and TOCNF/G sensors were measured. Compared with CNF, the TOCNF/G sensor exhibits the highest response and the fastest recovery. However, CNF sensor exhibited consistent response and recovery throughout the cycle (see Figure 6 and Figure 7). The change in electric resistance trend in both sensors was similar, in which resistance change was proportional to the analyte concentration, with the lowest limit of single-digit detection of ppm at 52% of relative humidity at room temperature. The gas response of the sensors was increased with increasing concentration of test gas, but the response curves were different (see Figure 6a and Figure 7a). Although TOCNF/G has more -OH groups than CNF, the TOCNF/G (5 MΩ) resistance was higher than CNF (0.25 MΩ), it may be due to CNF has an abundant porous surface structure (see Figure 4). One of the essential properties of a sensing material is porosity with a high surface area, which permits a fast analyte diffusion [53,54]. These properties of the CNF may support improving the ionic density on the surface of the sensor. A comparative histogram at 50 ppm of each test gas response towards the sensor is shown in Figure 6b and Figure 7b.

The calculated gas response values were 0.0101, 0.0095, 0.0059, and 0.0643 for the CNF sensor and 0.19000, 0.05095, 0.0309, and 0.0009 for the TOCNF/G sensor towards acetone, ammonia, methane, and hydrogen sulfide, respectively. The highest gas response was obtained towards hydrogen sulfide and acetone for the CNF and TOCNF/G sensor, respectively. However, the CNF sensor signal was not entirely recovered after exposure to hydrogen sulfide, which is attributed to the strong analyte interaction with CNF. The calculated response/recovery time toward acetone was 8.28/3.63 min and 1.45/3.39 min for the CNF and TOCNF/G sensors, respectively (see Figure 6c and Figure 7c). The TOCNF/G sensor took a shorter time to respond and recover compared to the CNF sensor. This might be due to the solid and uniform structure without pores in TOCNF/G sensors compared to CNF sensors, as confirmed by SEM analysis (see Figure 4). It is expected that the analyte absorption took place on the surface and the change of surface morphology could influence the response/recovery time of the sensors. The porous and rough surfaces in CNF take more time to absorb and diffuse analytes, thus taking more time to respond and recover. The comparative gas sensing performance of the sensors against the tested gases is presented in Table 1.

Nasution et al. fabricated chitosan/carboxymethylcellulose (CMC) acetone sensors in which sensors of pure chitosan and chitosan with a lower concentration of CMC composite only took 3 min for recovery. In comparison, the sensors with a higher CMC concentration required a longer time to recover; this is due to the uneven film surface, which allows the retained acetone to evaporate from the sensor surface [55]. Similarly, the results indicated that the measured resistance decreases significantly as the sensor is exposed to an increasing analyte concentration. Rahman et al. fabricated cellulose acetate-based nanofibers and nanofilms for H_2_S gas sensing applications at room temperature; they found that the current is increased when the sensor is exposed to an increasing concentration of H_2_S [49]. Similar results were obtained by Hittini et al. for a cellulose-copper oxide hydride nanocomposite membrane used for H_2_S gas detection, in which sensors exhibited high sensitivity and fast response to H_2_S at low temperature [24]. They also found that the sensors were selective for H_2_S and showed low humidity dependence.

When the sensors are exposed to an air atmosphere, oxygen molecules are absorbed on the surface of cellulose chains and eventually form oxygen-containing ions (e.g., O^−^, and O_2_^−^). These absorbed oxygen molecules lead to the formation of electron depletion layers. Li et al. also found that a surface enhancement with palladium-decorated gallium nitride (GaN) nanowire prepared for room-temperature methane gas sensors. They found that when CH_4_ molecules react with the oxygen ions of the surface of GaN nanowires, the oxidation-reduction reactions release the electron back to the conduction bands, so the electron depletion layers’ thickness and the resistance of the sensor decrease [56].

To further assess our prepared sensors’ gases and humidity-sensitive performance, some representative works are listed in Table 2. It can be found that prepared nanocellulose derived from rice husk sensors exhibits excellent performance with high sensitivity for various gases. However, it has a relatively long response and recovery time. This is mainly because of the massive number of hydroxyl groups in the cellulose chains that take a longer time to adsorb water molecules to reach saturation [21]. It is interesting to note that it is the only gas sensor among the reported sensors with the sensing capability to respond to a wide range of gases at room temperature at a relative humidity of 52% without doping. However, the sensors’ sensing performance may vary at different humidity conditions due to the hygroscopic properties of cellulose. The study is in the early stage; cyclic stability and hysteresis study are recommended at different humidity conditions and temperatures for further validation.

## 4. Conclusions

Monolithic or nanocomposite CNF gas sensors were fabricated by a facile method using rice husks as a cellulose source. After surface modification with TEMPO-mediated oxidation and glycerol, the as-prepared CNF and TOCNF/G sensors exhibited good gas sensing capability against acetone, ammonia, methane, and hydrogen sulfide in a humid environment at room temperature without doping. It is found that the introduction of abundant carboxyl and hydroxyl groups was vital for the change of ionic conductivity of the sensors upon gas exposure. The as-prepared CNF sensors may be suitable for developing a highly responsive nanocellulose-based gas sensor device. Although this study is still at the development stage, our results show the great potential of nanocellulose as a sensing material, which can be useful for onsite monitoring and detection of water-soluble gases in various applications. For example, they might be useful to determine the freshness of foods by early detection of gases formed during food deterioration.

## Figures and Tables

**Table 1:** Gas sensing performance of the CNF and TOCNF/G sensor.

Sensor	Response	Low Detection Level (ppm)	Response Time (S)	Recovery Time (S)	Response High (Low)
CNF	All gases	1 to 5	8.28	3.63	H_2_S (CH_4_)
TOCNF/G	All gases except H_2_S	1 to 10	1.45	3.39	Acetone (H_2_S)

**Table 2:** Comparison of the gas/humidity sensors performance between this work and previous publications.

Sensor Type	Output Signal	Relative Humidity	Response/Recovery Time	Ref.
Paper-based humidity and gas (H_2_S)	Resistance	45%	3 min	[57]
Perovskite halide on a paper (NH_3_)	Current	20 to 80%	~250 s (10 ppm) and ~300 s (50 ppm)	[58]
Nanostructured ZnO on cotton fabric	Resistance	51 ± 2%	58 s/85 s (NH_3_)	[59]
Proline ionic liquid/cellulose acetate	Resistance	NA	60 s/78 s (NH_3_)	[25]
Cellulose nanofibers/carbon nanotubes composite films-humidity sensor	Current	11 to 95%	330 s/377 s	[21]
Silicon-cellulose nanocrystal film-humidity sensor	Current	8 to 83%	0.04 s/0.04 s	[60]
Cellulose nanofibril/poly (ethylene glycol); with PEG/without PEG-humidity sensor	Impedance	20 to 90%	4.4/3.3 min 8.3/17 min	[33]
CNF and TOCNF/G multiple gases	Resistance	52%	8.3/3.6 min 1.4/3.4 min	our work

## Supplementary Materials

The following are available online at https://www.mdpi.com/article/10.3390/s21134415/s1, Table S1: XRD data with samples corresponding to 2 theta values. Figure S1: Comparative FTIR spectra of CNF, TEMPO-oxidized cellulose (TOCNF), and TOCNF with the addition of glycerol (TOCNF/G) in films.
