# Supplementary material for: Rice Husk-Derived Cellulose Nanofibers: A Potential Sensor for Water-Soluble Gases
Source: Sensors (Basel). 2021 Jun 28;21(13):4415. doi: 10.3390/s21134415 (PMC8271963; doi:10.3390/s21134415)
Supplement: Supplementary file 1 [file sensors-21-04415-s001.zip › sensors-1240235-supplementary.pdf]

Supporting Information (SI)

Rice Husk-Derived Cellulose Nanofibers: A Potential Sensor for Water-Soluble Gases

Naresh Shahi[1], Eunji Lee[2], Byungjin Min[3],* and Dong-Joo Kim[2],*

[1]College of Agriculture, Environment and Nutrition Sciences, Tuskegee University, Tuskegee, AL 36088 USA; nshahi1164@tuskegee.edu
[2]Department of Mechanical Engineering, Materials Research and Education Center, Auburn University, Auburn, AL 36849, USA; ezl0020@auburn.edu
[3]Department of Food and Nutritional Sciences, Tuskegee University, Tuskegee, AL 36088, USA
*Correspondence: bmin@tuskegee.edu (B.M.); Tel.: +1(334)-727-8393 (B.M.); dkim@auburn.edu (D.-J.K.); Tel.: +1(334)-844-4864 (D.-J.K.)

Table S1. XRD data with samples corresponding to 2 theta values.

Sample	CrI (%)
Cellulose nanofibers-rice husk (CNF)	53.39
TEMPO-Oxidized nanocellulose with glycerol (TOCNF/G)	59.51
TEMPO-oxidized nanocellulose (TOCNF)	65.12

The crystallinity index (CrI) was determined based on the reflected intensity data using the following equation.

$CrI\ (\%) = (I_{002} - I_{am})/I_{002} \times 100$

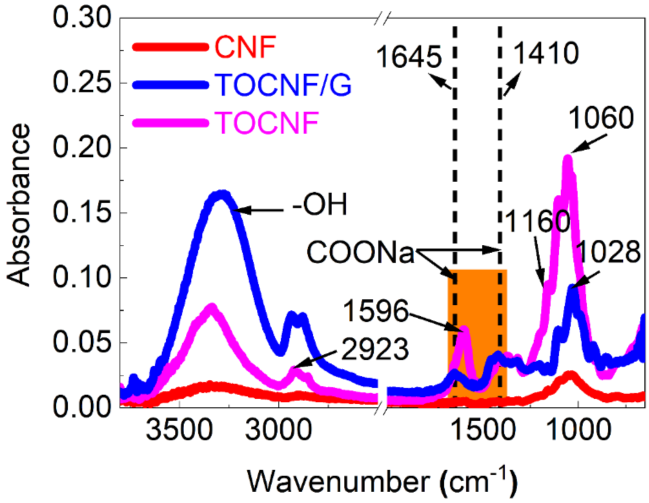

Figure S1. Comparative FTIR spectra of CNF, TEMPO-oxidized cellulose (TOCNF), and TOCNF with the addition of glycerol (TOCNF/G) in films. TOCNF/G has an intense peak around 3200-3300 cm^{-1} and is associated with -OH groups and a unique peak around 1645 and 1410 cm^{-1}; these peaks are associated with sodium carboxylate groups of TOCNF and TOCNF/G.
